# Effectiveness of Intermittent Preventive Treatment with Sulfadoxine-Pyrimethamine in Pregnancy: Low Coverage and High Prevalence of *Plasmodium falciparum* dhfr-dhps Quintuple Mutants as Major Challenges in Douala, an Urban Setting in Cameroon

**DOI:** 10.3390/pathogens12060844

**Published:** 2023-06-19

**Authors:** Carole Else Eboumbou Moukoko, Loick Pradel Kojom Foko, Angèle Ayina, Bernard Tornyigah, Annie Rachel Epote, Ida Calixte Penda, Patricia Epee Eboumbou, Serge Bruno Ebong, Gaetan Texier, Sandrine Eveline Nsango, Lawrence Ayong, Nicaise Tuikue Ndam, Albert Same Ekobo

**Affiliations:** 1 Malaria Research Unit, Centre Pasteur Cameroon, Yaoundé P.O. Box 1274, Cameroon; 2 Department of Biological Sciences, Faculty of Medicine and Pharmaceutical Sciences, The University of Douala, Douala P.O. Box 24157, Cameroon; 3 Laboratory of Parasitology, Mycology and Virology, Postgraduate Training Unit for Health Sciences, Postgraduate School for Pure and Applied Sciences, The University of Douala, Douala P.O. Box 24157, Cameroon; 4 ICMR-National Institute of Malaria Research, Dwarka, New Delhi 110077, India; 5 Pharmaceutical Sciences Department, Faculty of Medicine and Pharmaceutical Sciences, The University of Douala, Douala P.O. Box 24157, Cameroon; 6 Department of Parasitology, Noguchi Memorial Institute for Medical Research, College of Health Sciences, University of Ghana, Legon, Accra P.O. Box 1181, Ghana; 7 UMR 261 MERIT, Institut de Recherche pour le Développement (IRD), Université de Paris, 75006 Paris, France; 8 Haematology Laboratory, Centre Pasteur Cameroon, Yaoundé P.O. Box 1274, Cameroon; 9 Clinical Sciences Department, Faculty of Medicine and Pharmaceutical Sciences, The University of Douala, Douala P.O. Box 24157, Cameroon; 10 Pediatric Wards, Bonassama Hospital, Douala P.O. Box 9023, Cameroon; 11 Animal Organisms Biology and Physiology Department, Faculty of Sciences, The University of Douala, Douala P.O. Box 24157, Cameroon; 12 UMR 257-Vecteurs, Infections Tropicales et Méditerranéennes-VITROME-IRD/SSA/AP-HM, Aix-Marseille University, 13005 Marseille, France

**Keywords:** malaria infection, IPTp-SP, pregnant women, effectiveness, dhfr, dhps, k13 mutations, Cameroon

## Abstract

Intermittent preventive treatment in pregnancy with sulfadoxine and pyrimethamine (IPTp-SP) is a key component in the malaria control strategy implemented in Africa. The aim of this study was to determine IPTp-SP adherence and coverage, and the impact on maternal infection and birth outcomes in the context of widespread SP resistance in the city of Douala, Cameroon. Clinical and demographic information were documented among 888 pregnant women attending 3 health facilities, from the antenatal care visit to delivery. Positive samples were genotyped for *P. falciparum* gene (*dhfr*, *dhps*, and *k13*) mutations. The overall IPTp-SP coverage (≥three doses) was 17.5%, and 5.1% received no dose. *P. falciparum* prevalence was 16%, with a predominance of submicroscopic infections (89.3%). Malaria infection was significantly associated with locality and history of malaria, and it was reduced among women using indoor residual spraying. Optimal doses of IPTp-SP were significantly associated with reduced infection among newborns and women (secundiparous and multiparous), but there was no impact of IPTp-SP on the newborn bodyweight. *Pfdhfr*-*Pfdhps* quintuple mutants were over-represented (IRNI-FGKAA, IRNI-AGKAA), and sextuple mutants (IRNI-AGKAS, IRNI-FGEAA, IRNI-AGKGS) were also reported. The *Pfk13* gene mutations associated with artemisinin resistance were not detected. This study highlights the role of ANC in achieving optimal SP coverage in pregnant women, the mitigated impact of IPTp-SP on malaria outcomes, and the high prevalence of multiple SP-resistant *P. falciparum* parasites in the city of Douala that could compromise the efficacy of IPTp-SP.

## 1. Introduction

During pregnancy, especially in primiparous and secundiparous, women are at risk of several infectious diseases, including toxoplasmosis, leishmaniasis, and malaria [1,2]. Malaria in pregnancy (MiP) is an important public health concern in endemic areas, particularly in sub-Saharan Africa (sSA). In 2019, in the period of study, the World Health Organization (WHO) reported that 35% (~11.6 millions) of pregnancies in 33 sub-Saharan African countries with moderate-to-high risk transmission were exposed to malaria [3]. With the exception of *Plasmodium knowlesi*, all other species of human malaria parasites circulate in sSA, with *P. falciparum* being the predominant species [4]. Such infections can cause severe complications to the mother, her unborn baby, or child, and include maternal anemia, stillbirth, premature delivery, low birthweight, and growth retardation [5,6].

The management of MiP encompasses free distribution of long-lasting insecticide-treated nets, treatment of malaria cases, folic acid supplementation, and prevention of MiP through intermittent administration of sulfadoxine-pyrimethamine (SP), known as intermittent preventive treatment with SP during pregnancy (IPTp-SP) [3]. These strategies are implemented as part of antenatal care (ANC) services to prevent malaria and anemia in pregnant women living in moderate- to high-endemic areas. Since 2004, the WHO recommended IPTp-SP for preventing malaria episodes during pregnancy and for reducing the burden of MiP in sub-Saharan Africa, and to date, several malaria endemic countries have adopted IPTp-SP as a key preventive measure [3]. Many studies suggest a positive impact of IPTp-SP on maternal health and on morbidity and mortality in women and children [7,8,9]. However, considering the inadequacy of the two-dose regimen initially recommended to protect newborns from the deleterious effects of MiP during the third trimester, and that the beneficial effect of IPTp-SP on birthweight is dose-dependent [10,11], the WHO revised its policy in 2013. The revised WHO guidelines indicate that each pregnant woman should receive at least three doses of SP (IPTp-SP3+), also known as the optimal IPTp-SP dosage, with each dose administered at each ANC visit at one-month intervals, beginning in the second trimester and continuing until delivery [12].

In addition to the reported poor adherence and the low IPTp-SP uptake [13,14,15], many studies have highlighted that its effectiveness is reduced and compromised in areas where the prevalence of SP-resistant *P. falciparum* parasites is high in Africa [16], in the context where no other alternative drug with the same benefits as SP has yet been identified [17,18]. There is strong evidence that resistance to antimalarial drugs is associated with parasite genetic factors (single and crossed mutations in *P. falciparum dihydrofolate reductase* (*Pfdhfr*) and *dihydropteroate synthetase* (*Pfdhps*) genes). In this context, it is crucial to periodically monitor IPTp-SP coverage and effectiveness as well as the epidemiology of SP-resistant parasites among pregnant women in endemic areas, particularly in sSA which bears the bulk of the global MiP burden [3].

After the withdrawal of chloroquine and the adoption of the WHO policy in 2004, Cameroon have adopted and intensified the implementation of the revised WHO policy in 2013 with the aim to give at least three doses of SP between the 16th and the 36th weeks of gestation. Between 2014 and 2018, the proportion of pregnant women who received at least three doses of SP experienced a gradual progression, from 28% in 2014 to 38% in 2017, with regional disparities, and in the coastal region, this prevalence was estimated at 35% [19]. Cameroon is part of the WHO’s “High burden to High impact” strategy, which aims to reduce malaria by 2030 in the 11 highest-burden countries [20]. Research on the epidemiology of MiP, coverage, associated factors, and effectiveness of IPTp-SP is increasingly being conducted in Cameroon, particularly in the southwest, northwest, and central regions [21,22,23,24,25]. The present study was conducted in pregnant women living in the city of Douala, in the Littoral Region of Cameroon, where there is a paucity of data on the topic [26]. We determined the prevalence of malaria infection and submicroscopic parasitemia using quantitative polymerase chain reaction (qPCR), assessed the coverage and factors associated with IPTp-SP3+, and evaluated the effectiveness of IPTp-SP on selected maternal and birth outcomes. Additionally, *P. falciparum* malaria parasites were genotyped for mutations associated with SP and artemisinin (ART) resistance.

## 2. Materials and Methods

### 2.1. Study Sites

**Figure 1:**
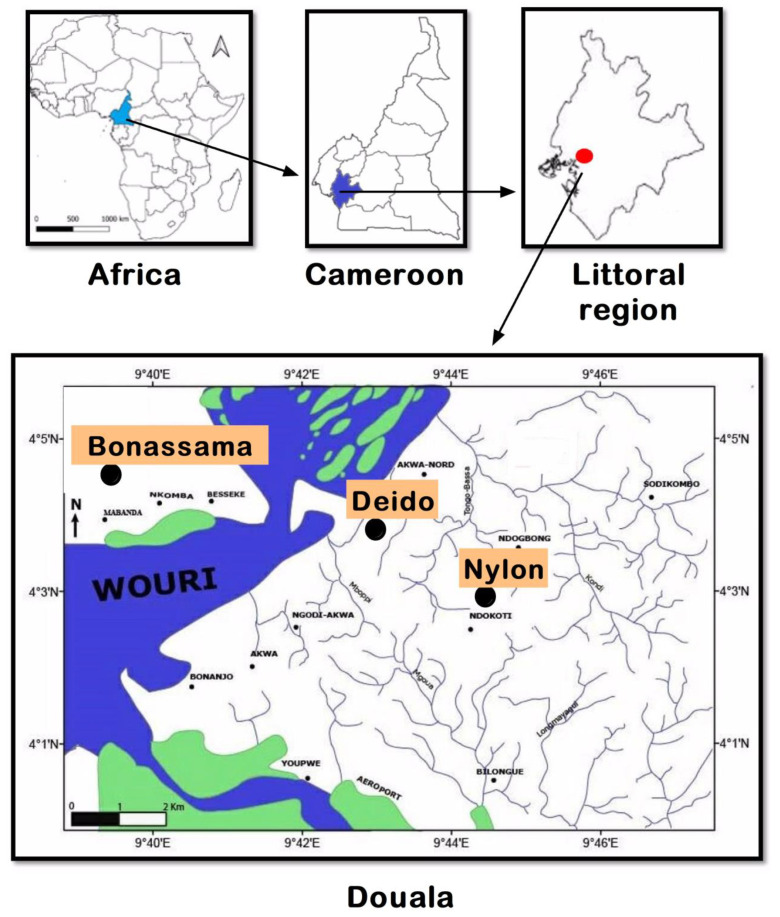
Location of the study sites in the town of Douala, Littoral Region, Cameroon. Note: Three health facilities were included in three health districts, referred to as the Deido, Bonassama, and Nylon districts, located in Douala III district of the Littoral Region in Cameron, Central Africa Region.

The study was conducted in the city of Douala (Littoral Region) (Figure 1). Douala is the economic capital of Cameroon, and it is divided into six districts (i.e., Douala I–VI districts). The population is greatly heterogeneous, with a predominance of three ethnic groups (*Duala*, *Bassa*, and *Bamileke*). The city is located in the tropical forest epidemiological area characterized by a diversity of ecosystems, heavy rainfall (1500–5000 mm^3^/year), and a humid climate. In this area, malaria transmission is perennial and holoendemic [27,28]. The main malarial species is *P. falciparum*, but other species including *P. ovale* and *P. vivax* have also been reported [29,30]. Three health facilities were included in the study, namely: (i) Deido District Hospital, located in the Douala I district, (ii) Bonassama District Hospital in the Douala IV district, and (iii) Nylon St Paul Maternity Clinic in the Douala III district. These three hospitals are represented as “Bonassama”, “Deido”, and “St Paul” in subsequent sections of this paper.

### 2.2. Study Design, Study Population, and Sampling Strategy

The study was designed as a hospital-based prospective survey and conducted from December 2015 to December 2016 in three facilities in Douala, Littoral Region, Cameroon. After obtaining ethical and administrative authorizations, pregnant women were approached to explain the objectives of the study, and informed consent was obtained. Sociodemographic, clinical, and paraclinical data were collected using an ad hoc survey form. Blood samples were collected for anemia and malaria diagnosis. Each participant was administered an SP dose as per national guidelines and received education on malaria and IPTp-SP.

The target population consisted of pregnant women attending the health facilities for antenatal care (ANC) visits and delivery. Women willing to participate in the study and having signed an informed consent form were included. Women infected with human immunodeficiency virus/under cotrimoxazole therapy, with a documented history of allergy to sulfamides and having recently taken SP alone or in combination with folic acid, were excluded from the study.

The women were consecutively recruited in each of the health facilities to limit selection and information biases. The sample size was computed using Lorentz’ formula: N = p × (1 − p) × z^2^/d^2^, where N is the required minimum sample size for the study, p (33%, as previously reported in the Littoral Region [31]) is the proportion of pregnant women reported to have received at least one IPTp-SP dose, z is the statistic for the desired confidence interval (z = 1.96 for the 95% confidence interval), and d is the accepted margin of error (d = 0.05). Thus, the minimum sample size was estimated to be 340 pregnant women.

### 2.3. Data Collection

A structured questionnaire consisting of five sections was used to collect data from each pregnant woman and their baby. The first section of the questionnaire captured sociodemographic information (age, region of origin, residence, level of education, occupation, and management of health expenses). The second part was designed to document knowledge on IPTp-SP and evaluate the environmental risk of malaria infection. The participants were asked about the presence of water collections (e.g., lake, bog), the implementation of a malaria-related prevention plan at home (i.e., insecticide-treated net, coils, insecticide residual spraying), and continuous education towards malaria. The third and fourth sections documented gynecological and family history, and clinical and physical information on ongoing pregnancy, respectively. In this section, information such as the date of the last menstruations, probable date of delivery, number of pregnancies, number of children, number of abortions, and comorbidities (diabetes, hypertension) was collected. The fifth section aimed to report data on biological examinations along with data on babies.

### 2.4. Blood Collection, Hematological, and Parasitological Analyses

About 4 mL of peripheral blood (women attending ANC and at delivery) and 2–4 mL of placental blood on the newborn side (newborn infection among women reporting for delivery at the hospital) were collected into EDTA tubes. The collected blood was used to perform biological tests. For molecular analysis, 160 µL of peripheral or placental blood was spotted onto blotting paper (Whatman FTA^®^ Elute) and stored at 4 °C until needed.

The hemoglobin (Hb) concentration and hematocrit (Hct) levels in the blood were determined using a portable hemoglobinometer (Hb Hemoglobin Test Strips, Mission^®^ Plus Hb, San Diego, CA, USA). Anemia in pregnancy was defined as an Hb level below 11 g/dL (or Hct < 33%) [32]. Anemia status was further classified into three categories (light, moderate, and severe) according to the most recent WHO criteria [32].

Malaria infection was determined using a rapid diagnostic test (Pan SD Bioline) and Giemsa-stained blood films (thick and thin smears). The Pan SD Bioline RDT is an immunochromatographic test that qualitatively detects the presence of the histidine-rich protein 2, an antigen specifically produced by *P. falciparum*, and pan lactate dehydrogenase that is produced by all *Plasmodium* species. This RDT is recommended by the WHO for malaria diagnosis [33], and was performed in accordance with the manufacturer’s instructions. Results were categorized as valid (testing positive or negative, and a positive control line) and invalid (absence of the control line). Blood films and microscopic examination were performed as previously described [34]. Thin blood films were used to establish malaria species, while thick blood films were used to quantify malaria parasitemia. Parasites were counted against at least 200 leucocytes, and parasitemia was determined by assuming a leucocyte density of 8000/mm^3^ for each participant [35]. Readings of the blood films were performed by skilled microscopists at the Centre Pasteur Cameroon.

### 2.5. Molecular Analysis: Parasite Detection by PCR and Genotyping of the Pfdhfr, Pfdhps, and Pfk13 Genes by Sequencing

All molecular analyses were performed at the Noguchi Memorial Institute for Medical Research (NMIMR), Accra, Ghana, and included quantitative polymerase chain reaction (qPCR) of *P. falciparum* samples and genotyping of drug resistance-associated *P. falciparum* genes. The plasmodial DNA was extracted from the dried blood spots using the Chelex method, as previously described [36].

Two quantitative polymerase chain reaction (qPCR) protocols were used to track *P. falciparum* infections, targeting the *var* gene acidic terminal sequence (varATS) and the telomere-associated repetitive element 2 (TARE2), respectively.

Parasitemia was quantified by extrapolation of cycle thresholds (Ct) from a standard curve of 3D7 *P. falciparum*-infected erythrocytes culture after 40–45 cycles for TARE2qPCR and 45 cycles for ATSqPCR. A negative control with no DNA template was run in all reactions. A threshold parasite density of >2 parasites/µL was considered positive [37]. Positive (3D7) and negative (distilled water) controls were run for each amplification. Submicroscopic infections were defined as infections not detected by microscopy examination but detected using qPCR. The presence of *P. falciparum* DNA in the extracted DNA was examined in duplicate by qPCR.

All samples that were detected positive by TARE2 qPCR where genotyped for three genes, namely dihydrofolate reductase (*Pfdhfr*) and dihydropteroate synthase (*Pfdhps*) genes associated with SP resistance, and the propeller domain of the Kelch 13 gene (*Pfk13*) associated with artemisinin (ART) resistance, as previously described [36,38,39]. PCR products detectable as a clear band of the expected sizes were purified using Wizard SV Gel and the PCR Clean-Up System^®^ (Promega, Madison, WI, USA) and sent for sequencing (GATC, Cologne, Germany) with the corresponding nested primers. The sequences generated were analyzed with the Chromas software [40], then aligned using the MEGA 5.2 software [41] and compared to the 3D7 *P. falciparum* reference genome. The SP resistance-associated mutations at positions 16, 51, 59, 108, and 164 in the *Pfdhfr* gene and positions 436, 437, and 540 in the *Pfdhps* gene were screened.

### 2.6. Statistical Analysis

Data were keyed into an Excel spreadsheet and then exported to StatView v5.03 for Windows (SAS Institute, Inc., Chicago, IL, USA) and GraphPad v7.03 for Windows (GraphPad PRISM, Inc., San Diego, CA, USA) for statistical analysis. Qualitative variables were presented as percentages, while quantitative variables were presented as mean ± standard deviation (SD). Pearson’s and Fisher’s exact tests were used to compare proportions. One-way analysis of variance and Student’s t-tests were used to compare mean values between ≥2 groups. Their non-parametric versions (i.e., Mann–Whitney and Kruskal–Wallis tests) were used as alternatives. Statistical significance was set at *p*-value < 0.05.

## 3. Results

### 3.1. Pregnant Women Included in the Study

**Figure 2:**
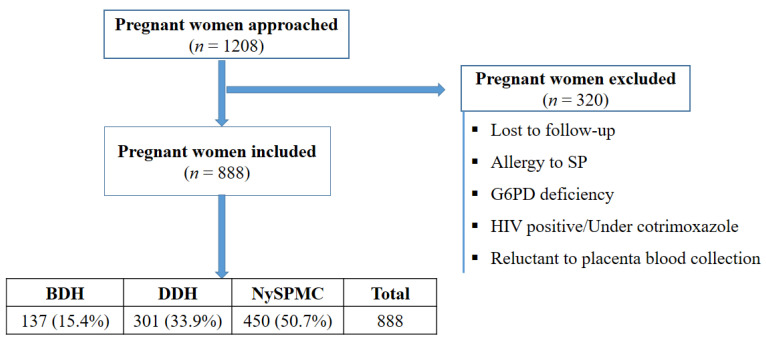
Flow diagram of the study depicting the process of participant inclusion. Note: SP: Sulfadoxine-pyrimethamine, G6PD: glucose-6-phosphate dehydrogenase, HIV: human immunodeficiency virus, BDH: Bonassama District Hospital, DDH: Deido District Hospital, NySPMC: Nylon St Paul Maternity Clinic.

A total of 1208 women were approached during the study, among them 888 were included based on different exclusion criteria (Figure 2).

### 3.2. Sociodemographic and Gynecological Characteristics

Women aged 22–26 and 26–31 years accounted for 28% and 32.9% of the participants, respectively. The overall mean age of the participants was 26.90 ± 5.30 years. More than half of the women had completed secondary education, and a statistically significant difference was found between the level of education and health facilities (Table 1). The proportion of primiparous women was significantly higher in women attending the Deido Hospital (43.5%) than among those attending the Bonassama (33.8%) and St Paul (31.3%) Hospitals. The overall proportion of women having received IPTp-SP during their previous pregnancies was 70.2%.

### 3.3. Antenatal Care Visits and IPTp-SP Coverage

**Figure 3:**
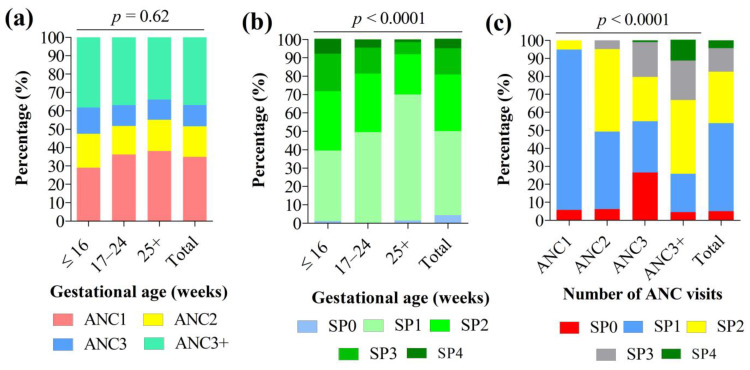
Association between (**a**) gestational age and antenatal care visits, (**b**) gestational age and IPTp-SP coverage, and (**c**) antenatal care visits and IPTp-SP coverage. Note: IPTp-SP: Intermittent preventive treatment with sulfadoxine-pyrimethamine in pregnancy. Pearson’s chi square test was used to compare percentages. Statistically significant at *p*-value < 0.05.

Analysis of the timing of the first ANC revealed that 29%, 36.2%, and 38.1% of women attended their first ANC at ≤16, 17–24, and 25+ weeks of pregnancy, respectively (Figure 3a). Women were 20.5 ± 4.9 weeks pregnant at their first ANC. No statistically significant association was found between gestational age and ANC visits (*p* = 0.62) (Figure 3a). At term, 5.1% (95% CI: 3.8–6.8%) of women had not yet received any dose of IPTp-SP. The IPTp coverage with one, two, three, or four doses of SP was 48.9% (95% CI: 45.5–52.1%), 28.5% (95% CI: 25.6–31.5%), 13.2% (95% CI: 11.1–15.6%), and 4.3% (95% CI: 3.2–5.9%), respectively. Thus, the overall proportion of women with adequate IPTp-SP coverage (at least three doses of SP, i.e., IPTp-SP3+) was 17.5% (95% CI: 15.1–20.2%).

The proportion of women attending their first ANC and receiving their first SP dose, and on time, was significantly higher in women with knowledge on IPTp-SP compared to their counterparts with no knowledge on IPTp-SP (27.4% vs. 18.7%, *p* = 0.04 for ANC, and 26.9% vs. 18.9%, *p* = 0.005 for SP dose). No statistically significant association was found between the timing of the first ANC, the first SP dose, and other participant characteristics (Supplementary File S1).

### 3.4. Factors Associated with Administration of at Least Three Doses of IPTp-SP (IPTp-SP3+) among Women at Delivery

Six determinants of optimal IPTp-SP coverage were identified based on multivariate logistic regression (participants’ age, level of education, number of ANC, timing of the first ANC, knowledge of IPTp-SP, and implementation of a prevention plan at home) (Table 2).

The uptake of the IPTp-SP optimal dose significantly increased with the level of education. Women having completed secondary and university studies had 21.61-fold (95% CI: 1.15–406.46, *p* = 0.007) and 6.30-fold (95% CI: 2.37–124.38, *p* = 0.002) higher uptake than their counterparts who had completed primary studies. An increase in age by one unit was associated with an increase in IPTp-SP3+ dose by 1.19 times (95% CI: 1.02–1.40, *p* = 0.03). Women having knowledge of IPTp-SP had three times more chance (OR = 2.91, 95% CI: 1.01–11.91, *p* = 0.04) to receive IPTp-SP3+ doses than those with no knowledge. Of note, the proportion of women with good knowledge of IPTp-SP significantly increased with the level of education, from 14.2% at the primary level to 26.2% at the university level (χ^2^ = 9.67, df = 3, *p* = 0.02). Interestingly, the chances of receiving optimal SP doses were reduced by 90% (OR = 0.10, 95% CI: 0.01–0.96, *p* = 0.04) in women attending their first ANC at ≥25 weeks of pregnancy compared to those attending at ≤16 weeks of pregnancy.

### 3.5. Prevalence of P. falciparum Malaria and Submicroscopic Infections in Peripheral Blood

**Figure 4:**
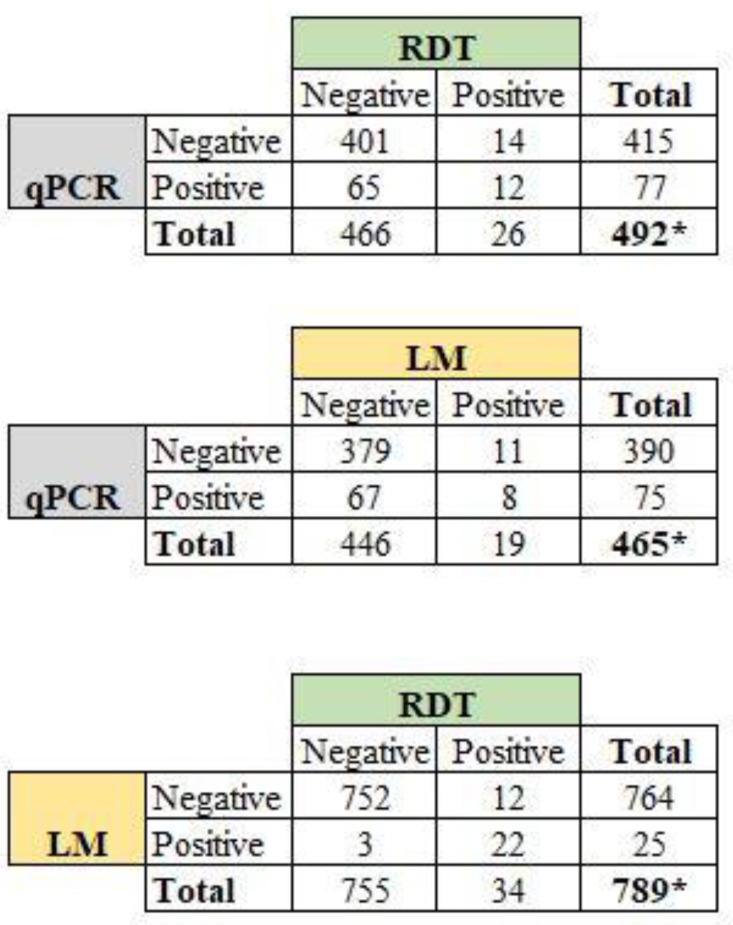
Proportion of peripheral *P. falciparum* infections among the women by comparing diagnostic methods. Note: LM: light microscopy; RDT: rapid diagnostic test; qPCR: quantitative polymerase chain reaction. * Total sample size was below 888 as results for LM, RDT, and qPCR were not available for some women during the study.

It should be noted that LM, RDT, and PCR results were not available for all women included in the study, for various reasons. We therefore calculated the prevalence of *P. falciparum* malaria for the techniques using different sample sizes (Figure 4). The *P. falciparum* prevalence rates were 3.2% (25/789, 95% CI: 2.2–4.6%) for LM, 4.2% (34/785, 95% CI: 3.1–5.9%) for RDT, and 16.0% (81/505, 95% CI: 13.1–19.5%) for qPCR. By comparing LM and qPCR, we have noted that 67 of 75 infections (89.3%) detected by qPCR were not detected by LM. Thus, the overall proportion of submicroscopic infections using peripheral blood was 14.4% (67/465, 95% CI: 11.5–17.9%) (Figure 4).

Considering only samples with available results of malaria infection for all three methods (n = 465 women), the prevalence of peripheral *P. falciparum* infection was 4.1% (95% CI: 31–5.5%) for LM, 5.6% (95% CI: 3.9–7.0%) for RDT, and 16.1% (95% CI: 8.7–12.9%) for qPCR.

### 3.6. Factors Associated with qPCR-Based P. falciparum Malaria Infection

Based on univariate logistic regression analysis, health facility, no knowledge of IPTp-SP, and history of malaria were associated with malaria infection (Table 3). After adjusting in the multivariate analysis, three factors were statistically associated with malaria infection. The odds of *P. falciparum* infection were 3.03 (95% CI: 1.02–8.85, *p* = 0.03) and 4.37 (95% CI: 1.52–12.27, *p* = 0.003) times higher in women attending the Deido and St Paul health facilities, respectively, compared to women attending the Bonassama Hospital. Women with a history of malaria were twice as likely (95% CI: 1.08–3.73, *p* = 0.02) to be infected with *P. falciparum* compared to those with no history of infection. The risk of peripheral *P. falciparum* infection was reduced by 47% in those using indoor residual (IRS) spraying (aOR: 0.53, 95% CI: 0.30–0.95, *p* = 0.03).

### 3.7. Anemia and Its Association with Malaria Infection, Submicroscopic Infections, and IPTp-SP

**Figure 5:**
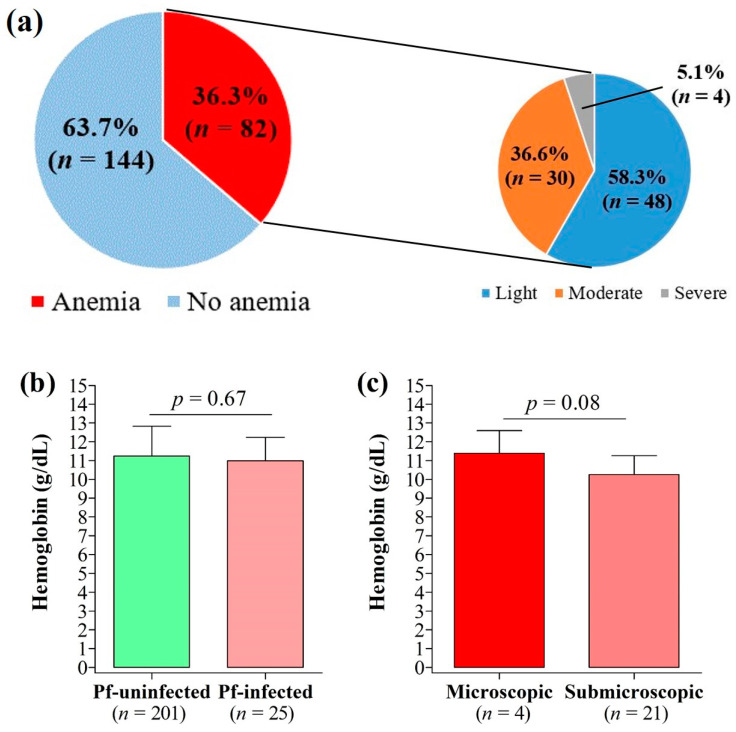
Anemia and its relation to malaria infection. (**a**) Prevalence of anemia, (**b**) variation of hemoglobin count by peripheral malaria infection, and (**c**) variation of hemoglobin count by type of peripheral malaria infection.

The prevalence of maternal anemia was 36.3%, with most cases graded as mild (58.5%) (Figure 5a). Hemoglobin levels were slightly lower in *P. falciparum*-infected patients compared to their uninfected counterparts, but the difference was not statistically significant (*p* = 0.67) (Figure 5b). Similarly, hemoglobin levels were statistically similar between women with microscopic infections and those with submicroscopic infections (*p* = 0.08) (Figure 5c). No statistically significant association was found between the prevalence of anemia or submicroscopic infections and the participant characteristics (health facilities, age groups, education level, marital status, occupation, implementation of prevention plan, IPTp-SP doses, ITN use, gestational age at first ANC, gestational age at first IPTp-SP dose, parity) (Supplementary File S2).

### 3.8. Impact of SP Dose on Maternal and Birth Outcomes

In total, 182 babies were included in the study, 2 in Bonassama, 52 in Deido, and 118 in St Paul health facilities. The overall proportion of low birthweight (LBW) was 9.34% (17/182, 95% CI: 5.9–14.5%). The proportion of LBW in Deido and St Paul health facilities was 5.8% (3/52, 95% CI: 1.9–15.6%) and 11.9% (14/118, 95% CI: 7.2–18.9%), respectively.

With respect to parity, the prevalence of maternal *P. falciparum* infection was significantly reduced with SP doses in secundiparous and multiparous women (Table 4). In secundiparous, the prevalence of anemia ranged from 33.3% in women having received no SP dose to 7.7% in those having received more than three doses (*p* = 0.02). In multiparous women, the prevalence ranged from 25% to 0% (*p* = 0.01). The IPTp-SP dose had no significant effect on maternal anemia, LBW, or neonatal *P. falciparum* infection.

### 3.9. Sulfadoxine-Pyrimethamine and Artemisinin Resistance Molecular Markers

Of the qPCR-positive samples, successful amplification of the *Pfdhfr*, *Pfdhps*, and *Pfk13* genes was achieved for 37, 35, and 32 samples, respectively (Table 5). Mutations 51I (83.3%), 59R (97.3%), and 108N (97.3%) in the *Pfdhfr* gene, and 437G (94.3%) in the *Pfdhps* gene, were predominantly found. Quadruple mutants were found in 6 (17.1%) of 35 sequenced samples, represented by NRNI-FGKAA, IRNI-AAKAA, and IRNI-FAKAA. Quintuple mutants (IRNI-AGKAA and IRNI-FGKAA) were the most represented of the multiple mutations, with 71.4% of samples. Three samples harbored the sextuple mutant and one a septuple mutant. There were no ART resistance-associated mutations in the *Pfk13* gene.

## 4. Discussion

Malaria due to *P. falciparum* continues to impose enormous losses in humans, especially pregnant women and children. The implementation of strategies such as IPTp-SP has greatly reduced the malaria burden in pregnant women. In this study, we assessed the level of IPTp-SP adherence and coverage as well as its determinants and its impact on maternal and neonatal outcomes in the city of Douala, Cameroon.

Knowledge of IPTp-SP was also associated with IPTp-SP3+ uptake, consistent with a previous study in the city of Bamenda, Northwest Cameroon [25]. This is likely due to the level of education as we found that a high proportion of women with knowledge of IPTp-SP had completed university studies. Several studies reported the positive impact of educational level on the optimal adoption of IPTp-SP in different settings [42,43,44,45]. The higher the level of education of women, the more likely they are to understand health messages about IPTp-SP during ANC or through other communication channels. We have previously shown the key role of educational level on other aspects of malaria, including knowledge, attitudes, and practices towards malaria and its prevention [46,47].

The present study supports the role of the number of ANC visits in the optimal coverage of IPTp-SP among pregnant women, and similar findings have been reported elsewhere [21,43,45,48,49]. Again, our study highlighted the crucial role of the timing of the first ANC visit in optimally covering pregnant women with IPTp-SP, and similar findings were reported in Tanzania and Uganda [43,45]. The earlier a woman attends the ANC visit, the more likely she is to receive the optimal dose of SP, as well as other preventive measures, such as long-lasting insecticide-treated nets and malaria awareness. Less than 20% of the ANC attendees had received an optimal SP dose, and this low coverage rate was similarly reported in previous studies conducted in Uganda and Tanzania [43,45,49]. Women at delivery had low coverage with IPTp-SP, and this is particularly worrying as it questions the availability of IPTp-SP in health facilities. Other studies reported higher rates of optimal IPTp-SP coverage [15,25], and the discrepancies between these studies and ours may likely be due to differences in the study designs, as these studies focused on women who had given birth recently or within a few years before the study.

The risk of *P. falciparum* malaria infection was low in women implementing IRS at home. This finding underscores the importance of implementing additional preventive methods, such as IRS, LLINs, and IPTp-SP. It is evident that the combination of different methods significantly reduces the chances of contracting malaria parasites, as found by Fokam and colleagues, who observed improved hemoglobin levels in pregnant women using both bed nets and IPTp-SP [23]. Malaria risk also varied by health facility. Most of the women lived close to the facility they attended, and thus the findings may reflect geographical variations in malaria risk [15,42,50].

More than five percent of the *P. falciparum* infections were submicroscopic, and this is similar to rates reported elsewhere [51]. Again, submicroscopic infections accounted for 60.2% of all infections, which is consistent with studies in malaria settings where submicroscopic parasitemia were found to be predominant [52,53,54,55,56,57,58,59,60]. Detection of submicroscopic infections is particularly challenging for efficient control, as they often constitute undetected parasite reservoirs [57]. Molecular methods are the gold standard for tracking these infections, but they are expensive and difficult to apply in the field in endemic countries, especially in Africa where resources are generally limited.

The results regarding the impact of IPTp-SP on maternal and birth outcomes were contrasted in this study as this preventive strategy reduced the risk of maternal infection but was not associated with maternal anemia and neonatal parameters. The literature on the impact of IPTp-SP is similar to ours, with divergent results on the subject [15,26,58,59]. Differences in malaria settings, study design, host characteristics, parasite characteristics, and host–parasite interactions could likely explain these contrasting findings on the impact of IPTp-SP on malaria burden.

IPTp-SP is still effective for the management of malaria in pregnant women in Cameroon [13,60]. Analyses of the *Pfdhfr*/*Pfdhps* genes identified the SP resistance-associated 59R and 108N mutations in *Pfdhfr* and 437G mutations in *Pfdhps* at rates close to 100%, and this is consistent with previous studies in Cameroon [61] and elsewhere [62]. The prevalence of the *Pfdhfr* 51I mutation (83.8%) found in this study was higher and not consistent with recent findings that showed that between 1980 and 2020, there was a significant decline in key mutations for *Pfdhfr* 51I (72.2–66.9%), *Pfdhfr* 59R (76.5–67.8%), and *Pfdhfr* 108N (90.8–67.6%), whereas the *Pfdhps* 437G mutation seems to be increasing over time (30.4–46.9%) [63]. In this study, we reported a higher level of the prevalence of 437G (94.3%). Both *Pfdhps* 540E and 581G mutations were found at rates < 5% among patients, lower than the prevalence where the WHO has recommended withdrawal of IPTp-SP, i.e., the prevalence of 581G > 10% and 540E > 95% [60]. Thus, our findings suggest the continuation of IPTp-SP use in MiP in Douala, but it should be interesting to conduct further studies with a higher number of *Pfdhfr*/*Pfdhps* sequences in the country to confirm these results. Nearly 89% of isolates carried the *Pfdhfr* IRN triple mutation, which is higher than that reported in one previous systematic review on mutation distribution in 2020 in Cameroon (~67.3%) and in Africa (~66% in 2020) [62,63]. In addition, quintuple mutants (I_51_R_59_N_108_-F_436_G_437_ and I_51_R_59_N_108_-A_436_G_437_) were predominantly found in isolates, and such mutants were previously reported in Cameroon and Ghana [61,64,65]. Interestingly, we noted that no wild-type at codon 436 was found in all isolates which were carrying either 436A or 436F. In African regions, most isolates carry S436 or 436A, and mutation 436F is generally found in low proportions [65].

We found *Pfdhfr*-*Pfdhps* sextuple mutants (I_51_R_59_N_108_-A_436_G_437_S_613_ and I_51_R_59_N_108_-F_436_G_437_E_540_) and septuple mutants (I_51_R_59_N_108_-A_436_G_437_G_581_S_613_) at prevalence rates below 11%. Such mutants, also known as fully resistant (I_51_R_59_N_108_-G_437_E_540_) and super resistant (I_51_R_59_N_108_-G_437_E_540_G_581_), confer a complete SP resistance and are strongly associated with treatment failures [66]. Only one isolate (2.9%) with five mutations associated with full resistance (i.e., I_51_R_59_N_108_-G_437_E_540_) was found in this study (this sample was otherwise with the F_436_ mutation), and this is in line with the previous low prevalence of this fully resistant mutation in Cameroon [61,63]. In Malawi and Myanmar, *P. falciparum* isolates with I_51_R_59_N_108_-G_437_E_540_ mutations are highly prevalent (>50%) [62]. Additionally, the I_51_R_59_N_108_-A_436_G_437_S_613_ sextuple mutant and the I_51_R_59_N_108_-A_436_G_437_G_581_S_613_ septuple mutant found at low rates in the present study have been reported in Cameroon, Ghana, Tanzania, and China [61,67,68,69]. In contrast, one study did not report quintuple, sextuple, or septuple mutants in febrile patients living in urban, semi-urban, or rural areas in Gabon [70]. Besides, these prevalence rates of quintuple and sextuple mutants could be underestimated considering the high fitness cost due to acquisition of such multiple mutation points, thereby reducing their density compared to susceptible strains in the absence of treatment. Thus, these multiple mutants likely could have been missed by detection methods in some patients due to the very-low-density parasites [70,71,72,73]. No mutation was identified in the sequenced *Pfk13* propeller gene.

## 5. Conclusions

In conclusion, coverage with optimal SP was low in pregnant women. A set of determinants of optimal IPTp-SP coverage was identified, with ANC being one of the most crucial, and the timing of the first ANC visit and the number of ANC visits were found to be strongly associated. Infection with *P. falciparum* was relatively high, with a predominance of submicroscopic infections. IPTp-SP positively affected malaria infection in women, but no noticeable effects of this preventive measure were found on maternal anemia and birth outcomes, such as LBW and neonatal infection. This study underscores the role of ANC in achieving optimal SP coverage in pregnant women. The high circulation rate of multiple SP-resistant *P. falciparum* parasites found in this study could likely explain the limited effect of IPTp-SP on maternal and birth parameters, and this could compromise not only IPTp-SP efficacy but also that of seasonal malaria chemoprevention with SP+ amodiaquine, now widely implemented and recognized as highly effective in protecting young children in the Sahelian Belt, north of Cameroon. The findings underscore the need for continuous monitoring of IPTp effectiveness in high-burden countries and call for urgent consideration of the need to revise national guidelines for the management of MiP.

## Figures and Tables

**Table 1:** Sociodemographic and gynecological characteristics of pregnant women.

Variables	Bonassama	Deido	St Paul	Total	*p*-Value
**Age groups**					
[14–17[	0 (0.0)	4 (1.4)	3 (0.7)	7 (0.9)	0.14
[17–22[	15 (31.2)	38 (13.0)	56 (12.5)	109 (13.8)	
[22–26[	11 (22.9)	84 (28.7)	126 (28.1)	221 (28.0)	
[26–31[	13 (27.1)	95 (32.3)	151 (33.7)	259 (32.9)	
[31–36[	6 (12.5)	51 (17.4)	82 (18.3)	139 (17.6)	
≥36	3 (6.3)	21 (7.2)	30 (6.7)	54 (6.8)	
**Level of education**					
None	1 (0.8)	0 (0.0)	3 (0.7)	4 (0.5)	0.005 *
Primary	19 (14.3)	32 (10.7)	69 (15.4)	120 (13.6)	
Secondary	92 (69.2)	169 (56.3)	264 (58.8)	525 (59.5)	
University	21 (15.7)	99 (33.0)	113 (25.1)	233 (26.4)	
**Marital status**					
Single	74 (60.2)	208 (69.3)	319 (71.1)	601 (68.9)	0.06
Married	49 (39.8)	92 (30.7)	130 (28.9)	271 (31.1)	
**Occupation**					
Agent	12 (8.9)	39 (13.2)	65 (14.7)	116 (13.3)	0.18
Top manager	12 (8.9)	29 (9.8)	27 (6.1)	68 (7.8)	
Student	31 (23.0)	88 (29.8)	103 (23.4)	222 (25.5)	
Housewife	36 (26.7)	70 (23.7)	120 (27.2)	226 (26.0)	
Unemployed	44 (32.5)	69 (23.5)	126 (28.6)	239 (27.4)	
**Health-related expenses**					
Insurance	0 (0.0)	1 (0.3)	1 (0.2)	2 (0.2)	1
Insurance + Personal	1 (0.8)	0 (0.0)	0 (0.0)	1 (0.1)	
Personal	131 (99.2)	299 (99.7)	448 (99.8)	878 (99.7)	
**IPTp-SP during previous pregnancies**					
No	40 (29.6)	68 (22.6)	155 (34.6)	263 (29.8)	0.002 *
Yes	95 (70.4)	233 (77.4)	293 (65.4)	621 (70.2)	
Parity					
Primiparous	45 (33.8)	131 (43.5)	140 (31.3)	316 (35.8)	0.006 *
Secundiparous	47 (35.3)	91 (30.2)	145 (32.4)	283 (32.1)	
Multiparous	41 (30.9)	79 (26.3)0	163 (36.3)	283 (32.1)	
**Number of children**					
0	45 (33.9)	131 (43.6)	140 (31.3)	316 (35.8)	0.01 *
1	47 (35.4)	91 (30.2)	145 (32.4)	283 (32.1)	
2	15 (11.2)	45 (14.9)	76 (16.9)	136 (15.4)	
3	20 (15.0)	18 (6.0)	57 (12.7)	95 (10.8)	
4	0 (0.0)	0 (0.0)	1 (0.2)	1 (0.1)	
≥4	6 (4.5)	16 (5.3)	29 (6.5)	51 (5.8)	
**Number of abortion events**					
0	78 (58.6)	175 (58.4)	269 (60.4)	522 (59.4)	0.04 *
1	32 (24.1)	97 (32.3)	113 (25.3)	242 (27.5)	
2	14 (10.5)	18 (6.0)	41 (9.2)	73 (8.3)	
3	8 (6.0)	4 (1.3)	18 (4.0)	30 (3.4)	
4	0 (0.0)	0 (0.0)	0 (0.0)	0 (0.0)	
≥4	1 (0.8)	6 (2.0)	5 (1.1)	12 (1.4)	

Data are number and proportion (%). * Pearson’s chi square and one-way analysis of variance (ANOVA) tests were used to compare percentage and mean values, and statistical significance was at *p*-value < 0.05. The total number (*n*) of pregnant women varies between 871 and 888 depending on the variables shown, due to missing data for some variables.

**Table 2:** Determinants of uptake of ITPp-SP3+ among full-term women.

Variables	N	*n* (%)	rOR (95% CI)	*p*-Value	aOR (95% CI)	*p*-Value
**Health facility**						
Bonassama	11	2 (18.2%)	1		1	
Deido	29	10 (34.5%)	2.37 (0.43–13.13)	0.32	1.06 (0.24–4.73)	0.93
St Paul	108	16 (14.8%)	0.70 (0.15–3.96)	0.76	0.60 (0.13–2.71)	0.51
**Age (years old)**	-	-	1.06 (0.98–1.15)	0.17	1.19 (1.02–1.40)	0.03 *
**Level of education**						
Primary	19	2 (10.5%)	1		1	
Secondary	78	16 (20.5%)	2.19 (0.45–10.49)	0.32	21.61 (1.15–406.46)	0.007 *
University	51	10 (19.6%)	2.07 (0.41–10.48)	0.37	6.30 (2.37–124.38)	0.002 *
**Marital status**						
Single	91	17 (18.7%)	1		1	
Married	57	11 (19.3%)	1.04 (0.45–2.42)	0.92	0.29 (0.07–1.19)	0.08
**Parity**						
Primiparous	313	59 (18.8%)	1		1	
Secundiparous	278	47 (16.9%)	0.88 (0.57–1.34)	0.54	0.38 (0.09–1.62)	0.19
Multiparous	279	47 (16.8%)	0.87 (0.57–1.33)	0.53	0.28 (0.04–1.74)	0.17
**Number of ANC**						
1	293	6 (2.0%)	1		1	
2	142	7 (4.9%)	2.48 (0.82–7.52)	0.11	1.61 (0.35–7.44)	0.54
3	100	29 (29.0%)	19.54 (7.81–48.86)	<0.0001 *	19.44 (6.36–59.43)	<0.0001 *
4+	313	104 (33.2%)	23.80 (10.25–55.25)	<0.0001 *	23.82 (2.01–282.25)	0.01*
No	123	20 (16.3%)	1		1	
Yes	25	8 (32.0%)	2.42 (0.92–6.38)	0.07	2.91 (1.01–11.91)	0.04 *
**History of malaria**						
No	106	20 (18.9%)	1		1	
Yes	40	8 (20.0%)	1.08 (0.43–2.68)	0.87	1.33 (0.40–4.39)	0.63
**Malaria prevention plan**						
No	20	2 (10.0%)	1		1	
Yes	128	26 (20.3%)	2.29 (0.50–10.52)	0.28	6.10 (0.55–67.58)	0.14
**Implementation of malaria prevention plan**						
No	34	8 (23.5%)	1		1	
Yes	110	20 (18.2%)	0.72 (0.29–1.82)	0.49	0.09 (0.02–0.52)	0.007 *
**Previous administration of IPTp-SP**						
No	37	7 (18.9%)	1		1	
Yes	111	21 (18.9%)	1.00 (0.39–2.59)	0.99	1.04 (0.23–4.80)	0.95
**Timing of the first ANC ^†^**						
≤16 weeks	30	7 (23.3%)	1		1	
17–24 weeks	91	19 (20.9%)	0.87 (0.32–2.32)	0.77	0.38 (0.09–1.50)	0.16
25+ weeks	24	2 (8.3%)	0.30 (0.06–1.60)	0.15	0.10 (0.01–0.96)	0.04 *

Data are number and proportion (%), unless otherwise indicated. ANC: antenatal care visit; IPTp: intermittent preventive treatment in pregnancy; SP: sulfadoxine-pyrimethamine; 95% CI: confidence interval at 95%; rOR: raw odds ratio; aOR: adjusted odds ratio. ^†^ We re-categorized this variable as previous categories had small sample sizes to compute the odds ratio. * Univariate and multivariate logistic regression were used to identify associated factors and statistical significance at *p*-value < 0.05.

**Table 3:** Univariate and multivariate logistic analysis of factors associated with qPCR-based peripheral *P. falciparum* infection among pregnant women.

Variables	N ^μ^	*n ^#^* (%)	rOR (95% CI)	*p*-Value	aOR (95% CI)	*p*-Value
**Health facility**						
Bonassama	137	4 (2.9%)	1		1	
Deido	301	25 (8.3%)	3.01 (1.03–8.83)	0.04 *	3.03 (1.02–8.85)	0.03 *
St Paul	450	52 (11.6%)	4.34 (1.54–12.24)	0.005 *	4.37 (1.52–12.27)	0.003 *
**Age groups**						
[14–17[	7	2 (28.6%)	1		1	
[17–22[	109	16 (14.7%)	0.43 (0.08–2.41)	0.34	0.60 (0.04–8.81)	0.71
[22–26[	221	19 (8.6%)	0.24 (0.04–1.30)	0.09	0.38 (0.03–5.61)	0.48
[26–31[	259	20 (7.7%)	0.21 (0.04–1.15)	0.07	0.33 (0.02–4.77)	0.41
[31–36[	139	14 (10.1%)	0.28 (0.05–1.58)	0.15	0.40 (0.03–6.41)	0.52
≥36	54	8 (14.8%)	0.43 (0.07–2.64)	0.37	1.61 (0.09–27.90)	0.74
**Marital status**						
Single	601	59 (9.8%)	1		1	
Married	271	22 (8.1%)	0.81 (0.49–1.36)	0.42	0.68 (0.34–1.37)	0.28
**Occupation**						
Agent	116	13 (11.2%)	1		1	
Top manager	68	3 (4.4%)	0.37 (0.10–1.33)	0.12	0.15 (0.02–1.33)	0.09
Student	222	23 (10.4%)	1.05 (0.52–2.11)	0.89	0.81 (0.31–2.16)	0.68
Housewife	226	14 (6.2%)	0.92 (0.45–1.88)	0.81	0.50 (0.20–1.30)	0.16
Unemployed	239	28 (11.7%)	0.52 (0.24–1.15)	0.11	0.92 (0.38–2.21)	0.84
**Parity**						
Primiparous	316	30 (9.5%)	1		1	
Secundiparous	283	26 (9.2%)	0.96 (0.56–1.67)	0.89	1.05 (0.50–2.23)	0.89
Multiparous	283	25 (8.8%)	0.92 (0.53–1.61)	0.78	0.86 (0.35–2.12)	0.74
**Number of ANC**						
1	300	29 (9.7%)	1		1	
2	143	11 (7.7%)	0.78 (0.38–1.61)	0.49	1.08 (0.37–3.13)	0.89
3	100	12 (12.0%)	1.27 (0.62–2.60)	0.51	2.36 (0.89–6.22)	0.08
4+	317	29 (9.1%)	0.94 (0.55–1.62)	0.83	1.62 (0.71–3.69)	0.25
**Received education on malaria**						
No	574	55 (9.6%)	1		1	
Yes	304	26 (8.6%)	0.88 (0.54–1.44)	0.61	1.23 (0.63–2.40)	0.54
**Knowledge of route of transmission**						
No	167	19 (11.4%)	1		1	
Yes	479	49 (10.2%)	0.89 (0.51–1.56)	0.68	1.35 (0.69–2.63)	0.38
**Knowledge of IPTp-SP**						
No	707	76 (10.7%)	1		1	
Yes	178	5 (2.8%)	0.24 (0.09–0.60)	0.002 *	0.30 (0.07–1.34)	0.11
**History of malaria**						
No	659	52 (7.9%)	1		1	
Yes	202	29 (14.4%)	1.96 (1.21–3.18)	0.006 *	2.01 (1.08–3.73)	0.02 *
**Presence of water collections around the house**						
No	403	31 (7.7%)	1		1	
Yes	479	50 (10.4%)	1.40 (0.88–2.24)	0.16	1.53 (0.72–3.25)	0.26
**Presence of swamp around the house**						
No	496	39 (7.9%)	1		1	
Yes	386	42 (10.9%)	1.43 (0.91–2.26)	0.13	0.94 (0.45–1.94)	0.86
**Outside Douala in the last 6 days**						
No	809	74 (9.1%)	1		1	
Yes	68	7 (10.3%)	1.14 (0.50–2.58)	0.75	1.26 (0.45–3.51)	0.65
**Implementation of malaria prevention plan**						
No	196	22 (11.2%)	1		1	
Yes	595	50 (8.4%)	0.73 (0.43–1.23)	0.23	0.69 (0.30–1.57)	0.37
**Previous administration of IPTp-SP**						
No	263	28 (10.6%)	1		1	
Yes	621	53 (8.5%)	0.78 (0.48–1.27)	0.32	0.99 (0.39–2.50)	0.98
**Utilization of ITNs**						
No	247	28 (11.3%)	1		1	
Yes	634	53 (8.4%)	0.71 (0.44–1.16)	0.17	0.85 (0.35–2.06)	0.71
**Indoor residual spraying**						
No	424	45 (10.6%)	1		1	
Yes	459	36 (7.8%)	0.72 (0.45–1.14)	0.16	0.53 (0.30–0.95)	0.03 *
**Aeration of sleeping place**						
No	713	65 (9.1%)	1		1	
Yes	161	16 (9.9%)	1.10 (0.62–1.97)	0.75	1.47 (0.74–2.92)	0.27

Data are number and proportion (%), unless otherwise indicated. ^μ^, Number of people in the group of variables studied; ^#^, number of infected participants. ANC: antenatal care visit; IPTp: intermittent preventive treatment in pregnancy; SP: sulfadoxine-pyrimethamine; ITN: insecticide-treated net; 95% CI: confidence interval at 95%; rOR: raw odds ratio; aOR: adjusted odds ratio in multivariate analysis. *, Significant *p*-value.

**Table 4:** Effect of IPTp-SP on maternal and birth parameters.

	IPTp-SP Doses					
Variables	0	1	2	3	3+	*p*-Value
**Maternal infection ^‡^**						
Primiparous	1 (6.3%)	18 (12.2%)	7 (7.8%)	3 (6.4%)	1 (8.3%)	0.69
Secundiparous	4 (33.3%)	9 (6.5%)	10 (12.5%)	2 (5.9%)	1 (7.7%)	0.02 *
Multiparous	4 (25%)	17 (12.4%)	3 (3.0%)	1 (2.9%)	0 (0.0%)	0.01 *
Overall	9 (20%)	44 (10.3%)	20 (8.0%)	6 (5.2%)	2 (5.3%)	0.03 *
**Maternal anemia**						
Primiparous	3 (75%)	12 (41.4%)	11 (37.9%)	4 (40%)	1 (33.3%)	0.68
Secundiparous	0 (0%)	8 (27.6%)	6 (28.6%)	2 (33.3%)	1 (50.0%)	0.88
Multiparous	2 (33.3%)	14 (37.8%)	11 (40.7%)	4 (40%)	1 (16.7%)	0.22
Overall	6 (40%)	34 (35.8%)	28 (36.4%)	10 (38.5%)	3 (27.3%)	0.22
**Newborn infection ^‡,#^**	2 (4.4%)	2 (5.3%)	0 (%)	0 (0%)	0 (0%)	0.03 *
**Low birthweight ^#^**	5 (11.1%)	4 (10.5%)	3 (5.6%)	2 (6.1%)	2 (16.7%)	0.78

Data are number and proportion (%), unless otherwise indicated. IPTp: intermittent preventive treatment in pregnancy, SP: sulfadoxine-pyrimethamine. ^‡^ *Plasmodium falciparum* parasites were detected in placental blood on the newborn side (newborn infection) using qPCR. ^#^ Variable was not adjusted on parity due to low sample size. * Pearson’s chi square test was used to compare percentages and was statistically significant at *p*-value < 0.05.

**Table 5:** Frequency of *Pfdhfr*, *Pfdhps*, and *Pfk13* genotypes among pregnant women.

Genes	wt/mt Codons *	Muted Alleles and Position ^†^	*n*	%
***dhfr* mutations** **(N = 37)**	AAT/ATT	51I	31	83.8
	TGT/CGT	59R	36	97.3
	AGC/AAC	108N	36	97.3
	ATA/TTA	164L	0	0.0
***dhps* mutations** **(N = 35)**	TCT/GCT	436A	14	40.0
	TCT/TTT	436F	21	60.0
	GCT/GGT	437G	33	94.3
	AAA/GAA	540E	1	2.9
	GCG/GGG	581G	1	2.9
	GCC/TCC	613S	3	8.6
***dhfr-dhps* mutations** **(N = 35)**	Quadruple mutants	NRNI-FGKAA	4	11.4
		IRNI-AAKAA	1	2.9
		IRNI-FAKAA	1	2.9
		IRNI-AGKAA	10	28.6
	Quintuple mutants	IRNI-FGKAA	15	42.9
		IRNI-AGKAS	2	5.7
	Sextuple mutants	IRNI-FGEAA	1	2.9
	Septuple mutant	IRNI-AGKGS	1	2.9
***k13* mutations (N = 32)**	-	Mutants	0	0

Data are number (*n*) and proportion (%) of isolates with mutations in the *Pfdhfr*, *Pfdhps*, and *Pfk13* genes. Mutations in nucleotide codons and amino acid sequences of the *Pfdhfr* and *Pfdhps* genes are underlined and in bold. *Pfdhfr*: *P. falciparum* dihydrofolate reductase, *Pfdhps*: *P. falciparum* dihydropteroate synthase. *, wt: Wild type, mt: mutant, ^†^ A: alanine, C: cysteine, E: glutamic acid, G: glycine, I: isoleucine, K: lysine, L: leucine, N: asparagine, R: arginine, S: serine.

## Data Availability

The datasets analyzed for this study are included in the article. Additional data required are available from the corresponding author upon reasonable request.

## Supplementary Materials

The following supporting information can be downloaded at: https://www.mdpi.com/article/10.3390/pathogens12060844/s1. Supplementary File S1: Timing of the first antenatal care visit, first dose of sulfadoxine-pyrimethamine, and the participants’ characteristics. Supplementary File S2: Prevalence of submicroscopic infections and anemia by patients’ characteristics.

## Abbreviations

ANC: antenatal care, ART: artemisinin, G6PD: glucose-6-phosphate dehydrogenase, Hb: hemoglobin, Hct: hematocrit, HIV: human immunodeficiency virus, IPTp: intermittent preventive treatment in pregnancy, IRS: indoor residual spraying, ITN: insecticide-treated net, LBW: low birthweight, LM: light microscopy, MiP: malaria in pregnancy, NMIMR: Noguchi Memorial Institute for Medical Research, qPCR: quantitative polymerase chain reaction, RDT: rapid diagnostic test, SD: standard deviation, SP: sulfadoxine-pyrimethamine, sSA: sub-Saharan Africa, TARE2: telomere-associated repetitive element 2, varATS: *var* gene acidic terminal sequence, WHO: World Health Organization.
