# Supplementary material for: Effectiveness of Intermittent Preventive Treatment with Sulfadoxine-Pyrimethamine in Pregnancy: Low Coverage and High Prevalence of Plasmodium falciparum dhfr-dhps Quintuple Mutants as Major Challenges in Douala, an Urban Setting in Cameroon
Source: Pathogens. 2023 Jun 19;12(6):844. doi: 10.3390/pathogens12060844 (PMC10300915; doi:10.3390/pathogens12060844)
Supplement: Supplementary file 1 [file pathogens-12-00844-s001.zip › pathogens-2376967-supplementary.pdf]

Supplementary file 1. Timing of the first antenatal care visit, first dose of sulfadoxine-pyrimethamine and the participants' characteristics

	First antenatal care visit								First dose of sulfadoxine-pyrimethamine							
	≤ 16 weeks		17–24 weeks		25+ weeks				≤ 16 weeks		17–24 weeks		25+ weeks			
**Variables**	**n**	**%**	**n**	**%**	**n**	**%**	**Decision variable**	**p-value**	**n**	**%**	**n**	**%**	**n**	**%**	**Decision variable**	**p-value**
**Site**																
Bonassama	29	22.5	78	60.5	22	17.0	7.58‡	0.11	28	21.7	84	65.1	17	13.2	8.81‡	0.06
Deido	68	25.2	153	56.7	49	18.1			69	25.4	155	57.0	48	17.6		
St Paul	74	17.0	281	64.4	81	18.6			71	17.1	272	65.5	72	17.4		
**Age (years old)**	26.94 ± 5.14		26.93 ± 5.13		26.83 ± 5.99		0.20†	0.98	26.99 ± 5.12		26.91 ± 5.17		26.66 ± 5.94		0.14†	0.86
**Level of education**																
None	1	25.0	3	75.0	0	0.0	9.35‡	0.15	1	25.0	3	75.0	0	0.0	11.81‡	0.06
Primary	23	20.5	64	57.1	25	22.4			22	20.6	61	57.0	24	22.4		
Secondary	88	17.7	319	64.2	90	18.1			85	17.6	319	65.9	80	16.5		
University	57	26.3	125	57.6	35	16.1			58	26.9	127	58.8	31	14.3		
**Marital status**																
Single	118	20.9	345	61.1	102	18.0	0.28‡	0.86	117	21.2	342	62.1	92	16.7	0.35‡	0.83
Married	50	19.5	158	61.5	49	19.0			49	19.4	159	63.1	44	17.5		
**Occupation**																
Agent	18	16.7	67	62.0	23	21.3	4.00‡	0.85	18	16.5	67	61.5	24	22.0	6.55‡	0.58
Top Manager	16	24.2	41	62.1	9	13.7			16	25.4	41	65.1	6	9.5		
Unemployed	45	19.7	137	60.1	46	20.2			43	19.5	138	62.7	39	17.7		
Student	43	21.0	126	61.5	36	17.5			43	21.1	127	62.3	34	16.6		

Housewife	48	22.6	129	60.8	35	16.6			47	23.0	126	61.8	31	15.2		
**Received education on malaria**														
No	103	19.0	336	62.0	103	19.0	2.61‡	0.27	101	19.3	332	63.4	91	17.3	1.88‡	0.38
Yes	67	23.5	171	60.0	47	16.5			66	23.3	172	60.8	45	15.9		
**Knowledge on IPTp-SP**														
No	125	18.7	419	62.6	125	18.7	6.28‡	0.04*	122	18.9	412	63.7	113	17.4	5.72‡	0.005*
Yes	45	27.4	93	56.7	26	15.9			45	26.9	99	59.3	23	13.8		
**History of malaria**														
No	122	19.4	396	62.9	112	17.7	2.66‡	0.26	119	19.5	392	64.3	99	16.2	3.63‡	0.16
Yes	42	22.7	104	56.2	39	21.1			43	23.4	104	56.5	37	20.1		
**Implementation of prevention plan at home**														
No	37	20.7	110	61.5	32	17.8	0.58‡	0.74	32	17.5	118	64.5	33	18.0	0.55‡	0.75
Yes	133	20.3	402	61.4	120	18.3			109	20.0	341	62.6	95	17.4		
**Parity**														
Primiparous	67	22.3	177	59.0	56	18.7	5.99‡	0.19	68	23.3	178	61.0	46	15.7	6.51‡	0.16
Secundiparous	62	22.9	162	59.8	47	17.3			59	22.6	160	61.3	42	16.1		
Multiparous	40	15.4	171	65.8	49	18.8			40	15.4	171	65.8	49	18.8		

IPTp-SP: Intermittent preventive treatment during pregnancy with sulfadoxine-pyrimethamine
Data are presented as frequency, percentage and mean ± standard deviation
Pearson’s independence chi square test was used to compare percentages while one-way analysis of variance was used to compare mean values
†Decision variable of the analysis of variance test
‡ Decision variable of the chi-square test
*Statistically significant at p-value < 0.05

Supplementary file 2. Prevalence of submicroscopic infections and anaemia by patients' characteristics

	Submicroscopic infections				Anemia			
**Variables**	**N**	***n* (%)**	**χ^2**	***p*-value**	**N**	***n* (%)**	**χ^2**	***p*-value**
**Health facilities**								
Bonassama	4	2 (50.0)	1.93	0.37	1	0 (0.0)	n.a	n.a
Deido	24	16 (66.7)			225	82 (36.4)		
St Paul	52	40 (76.9)						
**Age groups**								
[14 - 17[†	2	0 (0)	9.41	0.09	3	0 (0.0)	7.11	0.21
[17 - 22[	16	9 (56.3)			29	8 (27.6)		
[22 - 26[	19	15 (78.9)			60	18 (30.0)		
[26 - 31[	20	16 (80.0)			79	36 (45.6)		
[31 - 36[	13	10 (76.9)			41	14 (34.1)		
≥ 36	8	7 (87.5)			11	5 (45.5)		
**Level of education**								
None†	-	-	5.98	0.05	2	0 (0.0)	3.21	0.20
Primary	14	7 (50.0)			40	18 (45.0)		
Secondary	48	35 (72.9)			130	41 (31.5)		
University	18	16 (88.9)			53	22 (41.5)		
**Marital status**								
Single	58	41 (70.7)	-	0.77#	160	59 (36.9)	-	0.76#
Married	22	17 (77.3)			64	22 (34.4)		
**Occupation**								
Agent	13	13 (100)	7.71	0.11	30	9 (30.0)	4.25	0.37
Top manager	3	2 (66.7)			13	6 (46.2)		
Student	23	16 (69.6)			57	15 (26.3)		
Housewife	14	11 (78.6)			49	19 (38.8)		
Unemployed	27	16 (59.3)			71	29 (40.8)		
**Implementation of prevention plan**								
No	22	17 (77.3)	0.02	0.87	60	18 (30.0)	-	0.33#
Yes	49	37 (75.5)			150	57 (38.0)		
**Anemia**								
No	16	14 (87.5)	-	0.60#	-	-	n.a	n.a
Yes	9	7 (77.8)			-	-		
**IPTp-SP doses**								
Below 3	72	51 (70.8)	-	0.43#	187	68 (36.4)	-	0.99#
Three and above	8	7 (87.5)			37	13 (35.1)		
**ITN use**								
No	28	19 (67.9)	-	0.61#	66	20 (30.3)	-	0.28#
Yes	52	39 (75.0)			159	61 (38.4)		
**Gestational age at first ANC**								
≤ 16 weeks	15	12 (80.0)	1.02	0.59	40	13 (32.5)	0.19	0.91
17-24 weeks	45	30 (66.7)			146	52 (35.6)		

25+ weeks	15	11 (73.3)			35	13 (37.1)		
**Gestational age at first IPTp-SP dose**								
≤ 16 weeks	13	11 (84.6)	1.44	0.48	38	13 (34.2)	0.36	0.83
17-24 weeks	42	29 (69.0)			141	50 (35.5)		
25+ weeks	14	11 (78.6)			32	13 (40.6)		
**Parity**								
Primiparous	30	22 (73.3)	5.57	0.06	75	31 (41.3)	2.70	0.25
Secundiparous	26	15 (57.7)			64	18 (28.1)		
Multiparous	24	21 (87.5)			86	32 (37.2)		

ANC: Antenatal visit care, IPTp-SP: Intermittent preventive treatment in pregnancy with sulfadoxine and pyrimethamine, ITN: Insecticide-treated net, n.a: Not available, Data are number and proportion (%); †Excluded from statistical analysis, * Pearson's chi-square test and #Fisher's exact test were used to compare percentage, Statistical significance was set at p-value < 0.05.
